# A Local Energy Consumption Prediction-Based Clustering Protocol for Wireless Sensor Networks

**DOI:** 10.3390/s141223017

**Published:** 2014-12-03

**Authors:** Jiguo Yu, Li Feng, Lili Jia, Xin Gu, Dongxiao Yu

**Affiliations:** 1 School of Information Science and Engineering, Qufu Normal University, Rizhao 276826, Shandong, China; E-Mails: lilijia_qfnu@163.com (L.J.); guxinsd@sina.com (X.G.); 2 Faculty of Information Technology, Macau University of Science and Technology, Macau, China; E-Mail: lfeng@must.edu.mo; 3 Department of Computer Science, The University of Hong Kong, Pokfulam, Hong Kong, China; E-Mail: dxyu@cs.hku.hk

**Keywords:** wireless sensor networks, clustering, local energy consumption prediction, energy efficiency, network lifetime

## Abstract

Clustering is a fundamental and effective technique for utilizing sensor nodes' energy and extending the network lifetime for wireless sensor networks. In this paper, we propose a novel clustering protocol, LECP-CP (local energy consumption prediction-based clustering protocol), the core of which includes a novel cluster head election algorithm and an inter-cluster communication routing tree construction algorithm, both based on the predicted local energy consumption ratio of nodes. We also provide a more accurate and realistic cluster radius to minimize the energy consumption of the entire network. The global energy consumption can be optimized by the optimization of the local energy consumption, and the energy consumption among nodes can be balanced well. Simulation results validate our theoretical analysis and show that LECP-CP has high efficiency of energy utilization, good scalability and significant improvement in the network lifetime.

## 1. Introduction

A wireless sensor network (WSN) consists of plentiful low-power sensor nodes capable of sensing, processing and communicating. These sensor nodes observe the phenomenon at different points in the field, collaborate with each other and send the measured data to the base station (BS). Therefore, WSNs are extremely important in cyber-physical system (CPS) for observing and cognizing the complicated physical world at low cost [1]. However, sensor networks have limited and non-rechargeable energy resources; energy efficiency is a very important issue in designing the network topology, which affects the lifetime of sensor networks greatly. Thus, how to minimize energy consumption and maximize network lifetime are the central concerns when we design protocols for WSNs. Fortunately, these are the main goals of topology control [2,3]. Roughly speaking, topology control technology can be classified into two types. One is power control, and the other is hierarchical topology control. For the hierarchical topology control, generally, there exist about four methods, *i.e.*, clustering methods (e.g., [4–6]), connected dominating set methods (e.g., [7–11]), spanning tree methods (e.g., [12–14]) and spanner methods (e.g., [15,16]). By topology control, we usually can obtain a simplified topology of a given WSN while reserving connectivity (e.g., [17,18]) and coverage (e.g., [19–21]). Moreover, the diameter of the obtained topology cannot be increased any more (e.g., [22]).

As a kind of effective topology control method, clustering has proven to be an important way to decrease the energy consumption and to extend the lifetime of WSNs. In a clustering scheme, sensor nodes are grouped into clusters; in each cluster, one node is selected as the leader, named the cluster head (CH), and the other nodes are called cluster members (CMs). Each CM measures physical parameters related to its environment and then sends them to their CHs. When the data from all CMs is arrived, CHs aggregate the data and send it to the BS.

On the one hand, since CHs are responsible for receiving and aggregating the data from their CMs and transmitting the aggregated data to the specified destination, the energy consumption is much higher than that of CMs. Thus, to solve the problem, choosing appropriate cluster heads is the key issue when designing a cluster protocol. On the other hand, if CHs send the aggregated data to the BS directly, long-distance transmission will consume a lot of energy and lead to the premature death of CHs. Thus, designing a suitable inter-cluster multi-hop routing tree to forward data is also an important object of a clustering protocol.

In this paper, aiming at some energy heterogeneous WSNs where nodes are deployed uniformly, we propose a novel clustering protocol: LECP-CP (local energy consumption prediction-based clustering protocol), in which a new cluster head election algorithm is designed, which uses the predicted local energy consumption ratio of nodes as the parameter to compete for the role of the CH. Thus, the global energy consumption can be optimized by the optimization of the local energy consumption. To further reduce the energy consumption of CHs, we also propose a new inter-cluster communication routing tree construction algorithm, based on the local energy consumption ratio of nodes, as well. In addition, we provide explicit numerical calculations for the optimal cluster radius to minimize the energy consumption of the entire network, which is proven to be more accurate and realistic by theoretical analysis and simulation experiments.

The rest of the paper is organized as follows. Section 2 introduces the related works in this field. Section 3 gives the network model. Section 4 presents the local energy consumption prediction-based clustering protocol in details. Section 5 describes the derivation process of the local energy consumption ratio of nodes in detail. Section 6 analyzes several properties of our algorithms. Section 7 exhibits and analyzes our simulation results. Finally, Section 8 concludes the paper.

## 2. Related Work

In the choice of CHs, the criteria of existing clustering algorithms are different. We summarize the previous work about the selection methods of CHs as follows.

Low energy adaptive clustering hierarchy (LEACH) [23,24] randomly rotates the CHs to distribute the energy load among all of the sensor nodes in the network. The CHs' selection of them uses a probability scheme by which each node determines whether it is selected to be the CH based on the random number generated by itself. Although LEACH is simple and does not require a large communication overhead, it does not consider the energy and the distribution of CHs, which makes the algorithm energy-inefficient. Different from LEACH, DCHSintroduces the residual energy of nodes into the probability threshold, which improves the energy efficiency of the entire network and can extend the network lifetime effectively. Some other similar cluster head election algorithms based on the residual energy of nodes are also proposed in [25–29].

The algorithms proposed in [26,30,31] are the centralization version of LEACH. They improve LEACH by using central control, that is the BS collects information, such as node energy and location, from all sensor nodes and selects the optimal CHs. The defects of these are that the clustering process may be very complex and can generate more overhead. Thus, the centralized clustering algorithm has poor scalability and is only suitable for small or medium-sized networks. For this reason, most of the effective clustering algorithms are all distributed.

Hybrid energy efficient distributed clustering (HEED) [32] is a distributed clustering algorithm, in which CHs are selected from the sensor nodes based on a certain probability related to a hybrid of energy and communication cost. Only sensor nodes with high residual energy and lower intra-communication costs can become CHs. Clusters generated by HEED are more well-distributed than LEACH. However, it cannot guarantee the optimal number of elected CHs and the network connectivity. A similar, but improved, clustering algorithm, EEDC, is proposed in [33], which can reduce the number of iterations and prolong the network lifetime efficiently.

An energy-aware data gathering protocol for wireless sensor networks ( EADEEG) [34] is a novel distributed clustering algorithm. It elects cluster heads based on the ratio between the average residual energy of neighbor nodes and the residual energy of the node itself, which can achieve a good CH distribution and prolong the network lifetime. However, in some cases, there are “isolate points” in EADEEG, which influence the monitoring performance and lifetime of networks. In addition, it chooses 2*R_a_* as the inter-cluster communication radius, where *R_a_* denotes the cluster radius, which cannot ensure the connectivity among CHs. Similar to EADEEG, but improved, cluster head election algorithms are adopted in the cluster setup phase of clustering algorithms [35–37], which can effectively solve the “isolate points” problem existing in EADEEG, and the CHs generated by them can cover all the nodes in the network. For the disconnected problem, two energy-efficient clustering algorithms, called a distributed algorithm of clustering technology based on parameters used for electing CHs (BPEC) [38] and energy-efficient cluster formation protocol (EECF) [39], can keep all of the CHs connected by choosing 3*R_a_* as the inter-cluster communication radius.

Besides the node degree, location and energy, there still exist many other considerable influencing factors when designing clustering algorithms, such as coverage, connectivity, mobility, and so on. Soro *et al.* in [40] proposed some good cluster head election techniques. However, this paper focuses on coverage preservation, while the energy consumption and network lifetime is on the back burner. As a consequence, many hybrid control algorithms are also widely used in CHs selection. For instance, in [41], energy, concentration and centrality are combined to optimize the CH selection in order to extend the network lifetime efficiently. Energy and local distance are used in cluster head election mechanism using fuzzy logic (CHEF) [42] to compute the probability of being selected as CHs. The clustering algorithms proposed in [43,44] are similar to CHEF. The distance of the cluster centroid, the residual energy of nodes and network flow are selected to compute the selection probability of the CH. multi-weight based clustering (MWBC) [44] is a clustering algorithm based on multiple factors, such as the degree, current energy, transmission power, link quality and relative position of nodes when selecting CHs, aiming at maximizing the network lifetime. Simulation results demonstrate that these algorithms can make a good selection of CHs, and the network lifetime , to varying degrees, can be extended. However, in these algorithms, the weights of different factors can only be determined by experience instead of the precise calculations, which leads to the instability of these algorithms and, thereby, affects the performance of the entire network.

Generally, some algorithms mentioned above, such as [24,26–28], can take full advantage of the high energy of nodes. Selecting some high-energy nodes to take on the CH role ensures that the energy consumption of these CHs is balanced. However, there often exist the following cases. If the cluster size is not well-controlled, on the one hand, the energy consumption of some high-energy nodes may be also higher; after a round, the residual energy of these high-energy nodes is less than of these low-energy nodes, and on this occasion, the advantages of these nodes are not obvious. On the other hand, if the energy consumption of some high-energy nodes is lower, in this case, there will be much energy left while some other nodes have been dead, which cause the the network energy to be wasted. That is, in these cases, these algorithms cannot guarantee the local energy consumption optimization, let alone the global energy consumption optimization. Thus, to avoid these situations, we propose the concept of the local energy consumption ratio and then introduce it into the cluster head election phase. Some other algorithms, such as [41–44], also overlook this influencing factor in the choice of CHs. These hybrid algorithms consider the local energy consumption ratio as a parameter for nodes to compete for CHs, which is also helpful.

If CHs send their data to the BS directly, energy consumption increases if CHs are far away from the BS. Accordingly, for communication among CHs, searching for different communication routing paths among CHs to alleviate the loads of the CHs is also studied.

Applying the multi-hop communication method, each CH can find out the appropriate forwarding node, based on their distance to the BS, to relay data. The inter-cluster communication tree construction in energy-aware distributed unequal clustering (EADUC) [36] is based on the residual energy and relaying cost of forwarding nodes, while in EADC (energy aware distributed clustering) [37], the routing algorithm increases the forwarding tasks of the nodes in scarcely covered areas by forcing CHs to choose nodes with higher energy and fewer member nodes as their next hops, which can help EADC achieve load balance among CHs in non-uniform networks. For more results related to clustering protocols, one can refer to [5].

In this paper, to further reduce and balance the energy consumption of nodes, we construct a simple and energy-efficient inter-cluster communication tree based on the local energy consumption ratio of nodes in a distributed way.

## 3. Network Model

To simplify the network model, we adopt a few reasonable assumptions as follows.

(1) There are *N* sensor nodes that are distributed in an *M* × *M* square field.
(2) The BS and all of the nodes are stationary after deployment.
(3) All of the sensor nodes can be heterogeneous.
(4) All of the sensor nodes are location-unaware.
(5) All of the nodes can use power control to adjust the amount of transmit power.
(6) The BS is out of the sensor field. It has enough energy, and its location is known by each node.
(7) Each node has a unique identity *id*.

To transmit *l*-bit data to a distance *d*, the radio expends energy as:

(1) $$E_{Tx}\left(l,d\right)=\{\begin{array}{cc} l\times E_{\text{elec}}+l\times \varepsilon_{fs}\times d^{2}, & d<d_{0}\\ l\times E_{\text{elec}}+l\times \varepsilon_{mp}\times d^{4}, & d\geq d_{0} \end{array}$$

where *d* is the transmission distance. *E_elec_*, *ε_fs_* and *ε_mp_* are the parameters of the transmission/reception circuit. According to the distance between the transmitter and receiver, the free space (*ε_fs_*) or multi-path fading (*ε_mp_*) channel model is used.

While receiving *l*-bit data, the radio expends energy as:

(2) $$E_{Rx}=\left(l\right)=l\times E_{\text{elec}}.$$

## 4. LECP-CP Details

In this section, we give the details of LECP-CP. The whole operation is divided into rounds, and each round includes a cluster setup phase and a data transmission phase. To form a clustering topology, the cluster setup phase is divided into three subphases: node local energy consumption prediction phase, cluster head competition phase and cluster formation phase. In the data transmission phase, CMs collect the local data from the environment and send the collected data to the CHs. CHs receive and aggregate the data from their CMs and then send the aggregated data to the next-hop CH node based on the routing tree that we have constructed. The data transmission phase should be longer than the setup phase to reduce the overhead of the algorithm and to prolong the lifetime of the network. The state message of each node is listed in Table 1. Several control messages are needed, and the description of these messages is shown in Table 2.

### 4.1. Cluster Setup Phase

There are three subphases in the cluster setup phase: the node local energy consumption prediction phase, whose duration is *T*_1_; the cluster head competition phase, whose duration is *T*_2_; and the cluster formation phase, whose duration is *T*_3_.

#### 4.1.1. Node Local Energy Consumption Prediction Phase

When selecting CHs, LECP-CP firstly needs to predict the local energy consumption ratio of a node and then determines whether it has the ability to act as a CH.

Each node broadcasts a *Node_Msg* within radius *R_a_* with the following two values: the node *id* and its current energy *E_cur_*. At the same time, it receives the *Node_Msgs* from its neighbor nodes, each node can compute its approximate distance to its neighbor nodes based on the received signal strength; according to which, each node can predict its local energy consumption and calculate its local energy consumption ratio. Depending on the ratio, each node can determine whether it has the ability to act as a CH or not. For any node *s_i_*, we define its local energy consumption *ratio*(*s_i_*) as follows:

(3) $$\begin{array}{cc} \text{ratio}\left(s_{i}\right) & =\overset{n+1}{\underset{i=1}{\text{∑}}}E_{\text{con}}\left(s_{i}\right)/\overset{n+1}{\underset{i=1}{\text{∑}}}E_{\text{cur}}\left(s_{i}\right)\\ & =\left\{6\left(n+1\right)E_{\text{elec}}+\left(n+1\right)\left(E_{\text{sen}}+E_{\text{com}}\right)+\left(21+n\right)\varepsilon_{fs}R_{a}^{2}\right\}l/\overset{n+1}{\underset{i=1}{\text{∑}}}E_{\text{cur}}\left(s_{i}\right). \end{array}$$

where *E_cur_*(*s_i_*) denotes the current energy of node *s_i_*, *E_con_*(*s_i_*) denotes the energy consumption of node *s_i_* and *n* is the number of nodes within radius *R_a_* of *s_i_*. The detailed derivation process of the local energy consumption ratio of nodes is offered in Section 5.

For each node *s_i_*, we give the following formula to calculate its waiting time *t_i_* for broadcasting a *Head_Msg*.

(4) $$t_{i}=\text{ratio}\left(s_{i}\right)\cdot T_{2}\cdot V_{r}.$$

where *V_r_* is a real value randomly distributed in [0.9, 1], which is introduced to reduce the probability that two nodes send *Head_Msgs* at the same time.

According to Formulas (3) and (4), we can see that the smaller the local energy consumption ratio of node *s_i_* is, the shorter the waiting time *t_i_* is; thus, it is easier to be selected as the CH.

#### 4.1.2. Cluster Head Competition Phase

After *T*_1_ expires, LECP-CP starts the cluster head competition phase. For any node *s_i_*, in this phase, if it receives no *Head_Msg* when time *t_i_* expires, it broadcasts a *Head_Msg* within radius *R_a_* to advertise that it will be a CH. In Formula (4), a random value *V_r_* is introduced to reduce the probability that two nodes send *Head_Msgs* at the same time. Furthermore, each node only broadcasts the *Head_Msg* within radius *R_a_*. Thus, the probability that multiple nodes in the adjacent competition area have the same waiting time is very low, which means that there are few collisions among *Head_Msgs* from multiple nodes. Otherwise, if it receives a *Head_Msg* from *s_j_*, it records the *id* of *s_j_* and its distance to *s_j_*, then gives up the competition and, finally, becomes a plain node. As a plain node, node *s_i_* can keep on receiving *Head_Msgs*.

#### 4.1.3. Cluster Formation Phase

**Figure 1.**
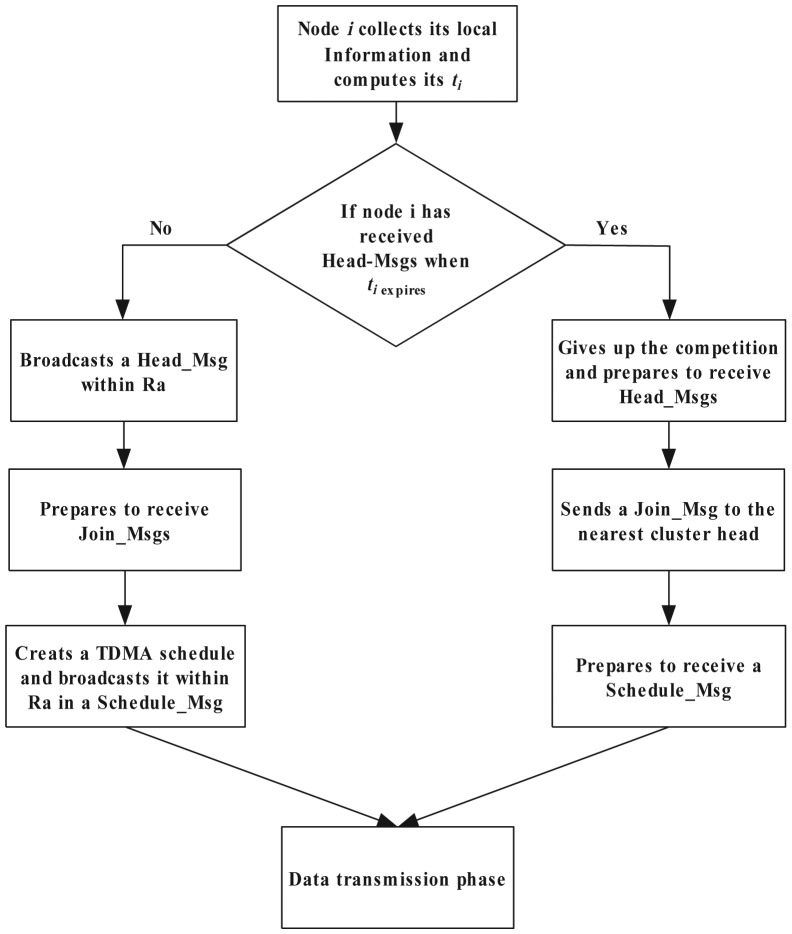
Cluster setup algorithm.

This is the last subphase of the cluster setup phase. Each plain node chooses the nearest CH and sends a *Join_Msg*, which contains its *id* and the current energy. Each CH creates a TDMA schedule list according to the received *Join_Msgs* and sends the schedule list to the CMs by broadcasting a *Schedule_Msg*. Each cluster is composed of the nodes in the Voronoi cell around the CH. Figure 1 illuminates the algorithm process of the cluster setup phase.

The following pseudo-code gives the details of the cluster setup phase.


**begin** (cluster setup algorithm) *state* ← *Candidate* Broadcast the *Node_Msg* **while** (*T*_1_ has not expired) **do**  Receive the *Node_Msg*  Update neighborhood table NT[ ]  *t_i_* ← broadcast waiting time **end** **while** (*T*_2_ has not expired) **do**  **if**
*CurrentTime* < *t_i_*
**do**   **if** receive a *Head_Msg* from the neighbor   NT[*i*] **do**    *state* ← *Plain*    NT[*i*].*state* ← *Head*   **else**    Continue   **end**  **else if**
*state* = *Candidate*
**do**   *state* ← *Head*   *R_a_* ← competing radius   Broadcast the *Head_Msg*  **end** **end** **while** (*T*_3_ has not expired) **do**  **if**
*state* = *Plain&&* has not sent the  *Join_Msg*
**do**   Send the *Join_Msg* to the nearest CH  **else if**
*state* = *Head*
**do**   Receive *Join_Msgs*  **end** **end****end**


### 4.2. Data Transmission Phase

In the data collection phase, each CM collects local data from the environment periodically and then sends the data to the CH within its time slot according to the TDMA scheduling list to avoid collisions among the members in the same cluster. When the data from all of the member nodes have arrived, the CH aggregates the data and sends them to the BS. Thus, this section is divided into two subphases, intra-cluster communication and inter-cluster communication. CMs sense and collect local data from the environment and send the collected data to the CHs. This process is called intra-cluster communication. For simplification, CMs communicate with CHs directly, just like LEACH. In the inter-cluster communication phase, we will construct a routing tree on the elected CH set, and each CH will forward the data it has collected and aggregated from their CMs to the BS by other CHs. This multi-hop communication from CHs to the BS will further reduce and balance the energy consumption.

Several nodes need to be selected as child nodes of the BS from all of the CHs and communicate with the BS directly. Therefore, each CH determines whether to be selected as the child node of the BS depending on its distance to the BS according to a threshold Euclidean distance *DIST*. If the distance from the CH *s_i_* to the BS is less than *DIST*, *s_i_* communicates with the BS directly and sets the BS as its next hop. Otherwise, it communicates with the BS through a multi-hop routing tree.

The concrete process is as follows. We set the duration as *T*_4_. At the beginning, each CH broadcasts a *Route_Msg* message within the radius *R_r_* with the values of the *id*, the node energy consumption ratio and the distance to the BS. To ensure the connectivity of all CHs, we set the radius *R_r_* = 3*R_a_*. If the distance from the CH *s_i_* to the BS is less than *DIST*, it chooses the BS as its next hop. Otherwise, it chooses its next hop according to the received *Route_Msg*. The CH *s_i_* chooses the neighbor CH node with a lower *ratio* and closer to the BS as its next hop.

**Figure 2.**
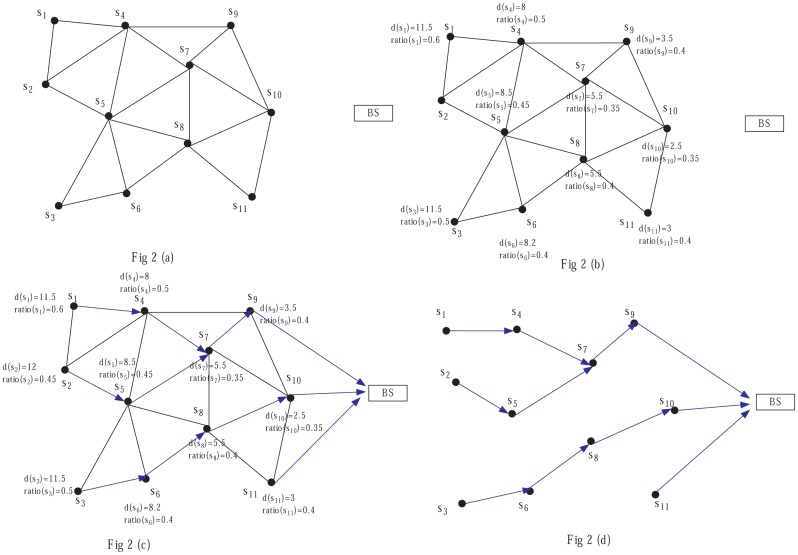
Construction of inter-cluster communication routing.

In the cluster setup phase, we adopt the same competition radius to construct clusters of even sizes; thus, the energy consumption among CMs can be balanced well, but the energy consumption of long distance transmission from CHs to BS is large; thus, we construct the routing tree among CHs with the method described above. For instance, in Figure 2, node *s*_1_ chooses its next hop CHs, which are closer to the BS than it; here, only *s*_4_ is chosen. For *s*_2_, when it chooses its next hop based on the distance to the BS, *s*_1_,*s*_4_,*s*_5_ are selected as candidate relay nodes; since *s*_5_ has the minimum ratio, *s*_5_ is finally selected. For *s*_4_, firstly *s*_7_ and *s*_9_ are selected, since *ratio*(*s*_7_) < *ratio*(*s*_9_), *s*_7_ is finally selected. For *s*_9_, *s*_10_ and *s*_11_, since their distances to the BS are smaller than *DIST*, they communicate with the BS directly.

The following pseudo-code gives the details of the data transmission phase.


**begin** (routing tree construction algorithm) Broadcast the *Route_Msg* **if**
*disttoBS* < *DIST*
**do**  *nexthop*← BS **else**  **while** (*T*_4_ has not expired) **do**  Receive the *Route_Msg*  Update CH neighborhood  table CHNT1[ ]  **end**  **if**
*s_j_* has the smaller value of disttoBS  in CHNT1[ ] **do**   update CHNT2[ ]  **end**  **if**
*s_k_* has the min ratio value in CHNT2[ ] **do**   *nexthop*← BS  **end** **end****end**


## 5. The Derivation Process of *Ratio*(*s_i_*)

When selecting the CH, LECP-CP firstly predicts the local energy consumption ratio of a node, then determines whether it has the ability to act as a CH. In this section, we analyze the local energy consumption ratio of any node *s_i_*.

In the cluster setup phase, a quantity of energy needs to be consumed. The energy expended on the clustering topology construction is called the additional energy cost, which includes the additional energy cost of a CH and many CMs in a cluster.

*E_a–ch_* and *E_a–cm_* are used to denote the additional energy cost of the CH and all CMs, respectively. In a round of the CH rotation process, a CH needs to broadcast one *Head_Msg*, one *Schedule_Msg* and one *Router_Msg*. It also needs to receive *n Join_Msgs* and one *Router_Msg*. The additional energy cost of the CH is as follows:

(5) $$\begin{array}{ll} E_{a–ch} & =2\left(E_{\text{elec}}+\varepsilon_{fs}R_{a}^{2}\right)l+\left[E_{\text{elec}}+\varepsilon_{fs}{\left(3R_{a}\right)}^{2}\right]l+nE_{\text{elec}}l+E_{\text{elec}}l\\ & =\left[\left(4+n\right)E_{\text{elec}}+11\varepsilon_{fs}R_{a}^{2}\right]l. \end{array}$$

In a round of the CH rotation process, since each node needs to send one *Join_Msg* and receive control messages from the CH, the additional energy cost of all CMs is as follows:

(6) $$\begin{array}{ll} E_{a–cm} & =n\left[2E_{\text{elec}}l+\left(E_{\text{elec}}+\varepsilon_{fs}d_{\text{toCH}}^{2}\right)l\right]\\ & =n\left(3E_{\text{elec}}+\varepsilon_{fs}d_{\text{toCH}}^{2}\right)l. \end{array}$$

where *d_toCH_* follows a uniform distribution over the interval [0, *R_a_*]. Consequently, the expected value of $d_{\text{toCH}}^{2}$ is:

(7) $$E\left[d_{\text{toCH}}^{2}\right]=\frac{R_{a}^{2}}{2}.$$

Now, substituting this in Equation (6), we have:

(8) $$E_{a–cm}=n\left(3E_{\text{elec}}+\varepsilon_{fs}\frac{R_{a}^{2}}{2}\right)l.$$

Therefore, the total additional energy of the cluster in a round of the CH rotation process is:

(9) $$\begin{array}{ll} E_{a–\text{total}} & =E_{a–ch}+E_{a–cm}\\ & =\left[\left(4n+4\right)E_{\text{elec}}+\left(11+\frac{n}{2}\right)\varepsilon_{fs}R_{a}^{2}\right]l. \end{array}$$

When the cluster setup phase expires, the data transmission phase begins. The energy consumption in this phase is called the effective energy cost. Each node collects the local data and sends it to the CH according to the TDMA scheduling list. When the data from all of the member nodes have arrived, the CH aggregates the data and sends them to its next-hop CH. This process is called a round of data collection. In each round, each cluster member needs to sense and send *l*-bit data to the CH. Let *E_e–cm_* denote the energy consumption of all CMs; we have:

(10) $$E_{e–cm}=n\left(E_{\text{sen}}+E_{\text{elec}}+\varepsilon_{fs}d_{\text{toCH}}^{2}\right)l.$$

where $E\left[d_{\text{toCH}}^{2}\right]=\frac{R_{a}^{2}}{2}$; thus, substituting it in Equation (10), we have:

(11) $$E_{e–cm}=n\left(E_{\text{sen}}+E_{\text{elec}}+\varepsilon_{fs}\frac{R_{a}^{2}}{2}\right)l.$$

where *E_sen_* is the energy used to sense the data. In each round, the effective energy consumption of the CH is:

(12) $$\begin{array}{ll} E_{e–ch} & =nE_{\text{elec}}l+E_{\text{sen}}l+\left(n+1\right)E_{\text{com}}l+2\left(E_{\text{elec}}+\varepsilon_{fs}d_{toNH}^{2}\right)l\\ & =\left[\left(n+2\right)E_{\text{elec}}+E_{\text{sen}}+\left(n+1\right)E_{\text{com}}+\varepsilon_{fs}d_{\text{toNH}}^{2}\right]l. \end{array}$$

where *d_toNH_* denotes the distance from the CH to its next-hop CH and *E_con_* is the energy used to aggregate the data from CMs. To ensure the connectivity of all CHs, we set the maximum inter-cluster communication radius *R_r_*=3*R_a_*, that is *d_toNH_* follows a uniform distribution over the interval [*R_a_*, 3*R_a_*]; thus, substituting this in Equation (12), we have:

(13) $$E_{e–ch}=\left[\left(n+2\right)E_{\text{elec}}+E_{\text{sen}}+\left(n+1\right)E_{\text{com}}+10\varepsilon_{fs}R_{a}^{2}\right]l.$$

Thus, the total effective energy consumption *E_e–total_* in a cluster is:

(14) $$\begin{array}{ll} E_{e–\text{total}} & =E_{e–ch}+E_{e–cm}\\ & =\left[\left(2n+2\right)E_{\text{elec}}+\left(n+1\right)\left(E_{\text{elec}}+E_{\text{sen}}\right)+(\left(10+\frac{n}{2}\right)\varepsilon_{fs}R_{a}^{2}\right]l. \end{array}$$

Here, we can obtain the total energy consumption in a cluster from Equations (9) and (14). We have:

(15) $$\begin{array}{ll} E_{\text{all}–\text{total}} & =E_{e–\text{total}}+E_{a–\text{total}}\\ & =\left\{\left(6n+6\right)E_{\text{elec}}+\left(n+1\right)\left(E_{\text{sen}}+E_{\text{com}}\right)+\left(21+n\right)\varepsilon_{fs}R_{a}^{2}\right\}l. \end{array}$$

By analyzing, we know:

(16) $$E_{\text{all}–\text{total}}=\overset{n+1}{\underset{i=1}{\text{∑}}}E_{\text{con}}\left(s_{i}\right).$$

Since $\text{ratio}\left(s_{i}\right)=\overset{n+1}{\underset{i=1}{\text{∑}}}E_{\text{con}}\left(s_{i}\right)/\overset{n+1}{\underset{i=1}{\text{∑}}}E_{\text{cur}}\left(s_{i}\right)$, which is introduced in Section 4.1.1, then we finally obtain Equation (3) when combining Formulas (15) and (16).

Obviously, for any *s_i_*, its local energy consumption ratio can be calculated in advance, and the *ratio*(*s_i_*) can be used to determine whether *s_i_* will be selected as the CH; additionally, we can draw the conclusion from Equation (3) that the more the total current energy and the fewer the energy consumption of nodes within the cluster radius of *s_i_* are, the smaller the *ratio*(*s_i_*) is. Thus, it is more accurate and realistic to make *ratio*(*s_i_*) the parameter of *s_i_* to compete for the role of the CH.

## 6. LECP-CP Analysis

**Theorem 1:** The CH set generated by LECP-CP can cover all of the network nodes, and there is at most one CH within the cluster radius *R_a_* of any CH.

**Proof:** *t_i_* = *ratio*(*s_i_*) · *T*_2_ · *V_r_* according to Formula (4). Thus, we can obtain *t_i_* < *T*_2_ since *ratio*(*s_i_*) < 1 and *V_r_* < 1. That is, the waiting time *t_i_* of any node *s_i_* is smaller than *T*_2_. Thus, any expected CH will broadcast a *Head_Msg* and become a CH before *T*_2_ expiring, which can avoid the generation of “isolate points”.

As we stated previously, Formula (4) ensures that different nodes have different waiting times. We assume that node *s_i_* has a shorter waiting time than others and broadcasts the *Head_Msg* within radius *R_a_*. Thus, all of the nodes within this range will give up the competition and become CMs. Therefore, there is no more than one CH within the radius *R_a_* of any CH.

**Lemma 1:** Given a maximal independent set *S* of an undirected connected graph *G*(*V,E*), if the number of nodes in *S* is no less than two, then there must exist at least one node in *S* within three hops of every node *υ* in *S*.

**Theorem 2:** If the inter-cluster transmission range *R_inter–CH_* and the intra-cluster transmission range *R_intra–CH_* satisfy that *R_inter–CH_* ≥ 3*R_intra–CH_*, then the CH set generated by LECP-CP algorithm is a connected dominating set of the network.

**Proof:** Firstly, we prove that the CH set *S* generated by LECP-CP algorithm is a dominating set. According to Theorem 1, there is no more than one CH within a cluster, so the CH set *S* must be an independent set. After the execution of the LECP-CP algorithm, each node in the network either is the CH, or the member node of one cluster, and any plain node adding to the cluster head set will destroy its independence; so, the CH set *S* is the maximum independent set. Since the maximum independent set is also a dominating set, the CH set generated by the LECP-CP algorithm is a dominating set of the network.

**Figure 3.**
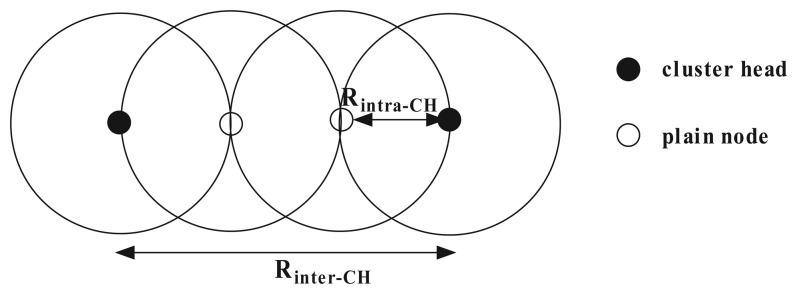
A case of inter-cluster and intra-cluster transmission range.

Then, we prove that CHs in the dominating set *S* are connected if the inter-cluster transmission range *R_inter–CH_* and the intra-cluster transmission range *R_intra–CH_* satisfy that *R_inter–CH_* ≥ 3*R_intra–CH_*. According to Lemma 1, there must exist at least one cluster head node in *S* within three hops of any CH *s_i_* of *S*. Figure 3 depicts a case exhibiting the longest distance between two adjacent CHs. In this worst-case configuration, it is clear that when the transmission range *R_inter–CH_* of a CH is not less than three-times *R_intra–CH_*, the overlay graph composed of CHs will be connected.

**Lemma 2:** Suppose that the network area is *A* and the cluster radius is *R_a_*, then the expected number of CHs generated in the network is $M_{\exp}=\left\lceil \frac{4A}{3\sqrt{3}R_{a}^{2}}\right\rceil$.

**Figure 4.**
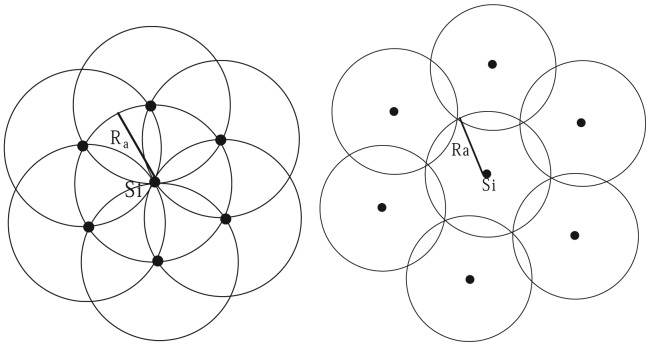
The maximum cluster area and minimum cluster area.

**Proof:** Clusters generated by the clustering algorithm need to cover all nodes in the network, that is they need to cover the whole network. Since there are overlaps among clusters and there is only one CH in any cluster, thus when the overlaps among clusters are the most, the number of CHs achieves the maximum; to the contrary, there is the least number of CHs in the network when the overlaps among clusters are the least. BPEC [16] shows the cases of minimum cluster area and maximum cluster area, depicted by Figure 4. On these occasions, we can correspondingly obtain the maximum number of CHs $M_{\max}=\left\lceil \frac{2A}{\sqrt{3}R_{a}^{2}}\right\rceil$ and the minimum number of CHs $M_{\min}=\left\lceil \frac{2A}{3\sqrt{3}R_{a}^{2}}\right\rceil$, respectively.

Since the maximum and minimum cluster area are both proportional to $R_{a}^{2}$, thus we can deduce that the cluster area in the practical network is also proportional to $R_{a}^{2}$. Take the condition where the heterogeneous nodes are randomly deployed in the network into consideration: the expected number of CHs in the practical network is taken as $M_{\exp}=\left\lceil \frac{4A}{3\sqrt{3}R_{a}^{2}}\right\rceil$.

**Theorem 3:** If *N* nodes are randomly deployed over a square field, the area of which is *A*, then when the cluster radius is set as the optimal value $R_{\text{opt}}=\sqrt[{4}]{\frac{4\sqrt{3}A\left(6E_{\text{elec}}+E_{\text{sen}}+E_{\text{com}}\right)}{9N\varepsilon_{fs}}}$, the energy consumption of the entire network can be minimized.

**Proof:** In Section 5, we have obtained that in a round of the CH rotation process, the total energy consumption in a cluster is:

(17) $$E_{\text{all}–\text{total}}=\left\{\left(6n+6\right)E_{\text{elec}}+\left(n+1\right)\left(E_{\text{sen}}+E_{\text{com}}\right)+\left(21+n\right)\varepsilon_{fs}R_{a}^{2}\right\}l.$$

Obviously, if the energy consumption in each cluster of one round is the minimum, then the total energy consumption in the network is the minimum. According to Lemma 2, the expected number of CHs in the practical network is taken as $M_{\exp}=\left\lceil \frac{4A}{3\sqrt{3}R_{a}^{2}}\right\rceil$; thus, the energy consumption of all clusters in one round is:

(18) $$\begin{array}{ll} E_{\text{total}} & =M_{\exp}E_{\text{all}–\text{total}}\\ & =M_{\exp}\left[\left(6n+6\right)E_{\text{elec}}+\left(n+1\right)\left(E_{\text{sen}}+E_{\text{com}}\right)+\left(21+n\right)\varepsilon_{fs}R_{a}^{2}\right]l\\ & =M_{\exp}\left[\left(\frac{N}{M_{\exp}}+1\right)\left(6E_{\text{elec}}+E_{\text{sen}}+E_{\text{com}}\right)+\left(21+\frac{N}{M_{\exp}}\right)\varepsilon fsR_{a}^{2}\right]l\\ & =\left[\left(6E_{\text{elec}}+E_{\text{sen}}+E_{\text{com}}\right)\left(N+\frac{4A}{3\sqrt{3}R_{a}^{2}}\right)+\frac{28A}{\sqrt{3}}\varepsilon_{fs}+N\varepsilon_{fs}R_{a}^{2}\right]l. \end{array}$$

Taking the derivative of Formula (18) over *R_a_* and making the derivation result be zero, then we can obtain the optimal cluster radius *R_opt_*:

(19) $$R_{\text{opt}}=\sqrt[{4}]{\frac{4\sqrt{3}A\left(6E_{\text{elec}}+E_{\text{sen}}+E_{\text{com}}\right)}{9N\varepsilon_{fs}}}$$

**Theorem 4:** The overhead complexity of control messages in the network is *O*(*N*).

**Proof:** At the beginning of each round, each node broadcasts a *Node_Msg*. Thus, there are *N Node_Msgs* in the whole network. In each round, each cluster member broadcasts a *Join_Msg*, while each CH broadcasts a *Head_Msg*, a *Schedule_Msg* and a *Route_Msg*. Suppose the number of generated CHs is *k*; then, the total number of *Join_Msgs* is *N* − *k*, and the numbers of *Head_Msgs*, *Schedule_Msgs* and *Route_Msgs* messages are all *k*. Thus, the total number of control messages in the entire network is *N* + (*N* − *k*) + *k* + *k* + *k* = 2*N* + 2*k*. Therefore, the overhead complexity of control messages in the network is *O*(*N*).

**Theorem 5:** The time complexity of LECP-CP is *O*(1).

**Proof:** LECP-CP adopts a distributed clustering strategy. Thus, the time complexity of the entire network is equal to that of a single node *O*(1). In other words, the time complexity is a constant and has nothing to do with the network size.

## 7. Experimental Section

The simulation was performed in *NS*-2. Every simulation result shown in this section is the average result of 50 independent experiments, unless otherwise specified. Each experiment is done in different scenarios where the nodes are uniformly deployed over a 200 m × 200 m field, and four scenarios (100 nodes, 200 nodes, 300 nodes, 400 nodes) are chosen.

**Figure 5.**
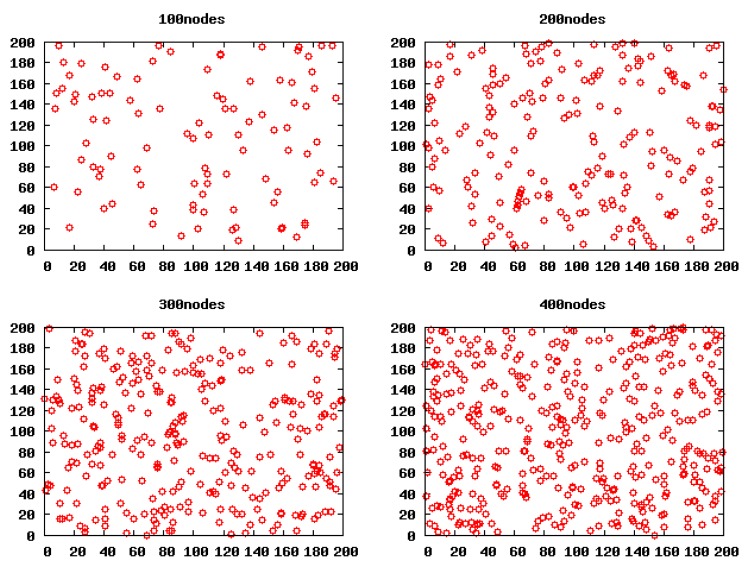
The initial network topology of the four scenarios.

Figure 5 shows the initial network topology of the four scenarios.

The parameters of the simulations are listed in Table 3.

### 7.1. Algorithm Properties Validation

**Figure 6.**
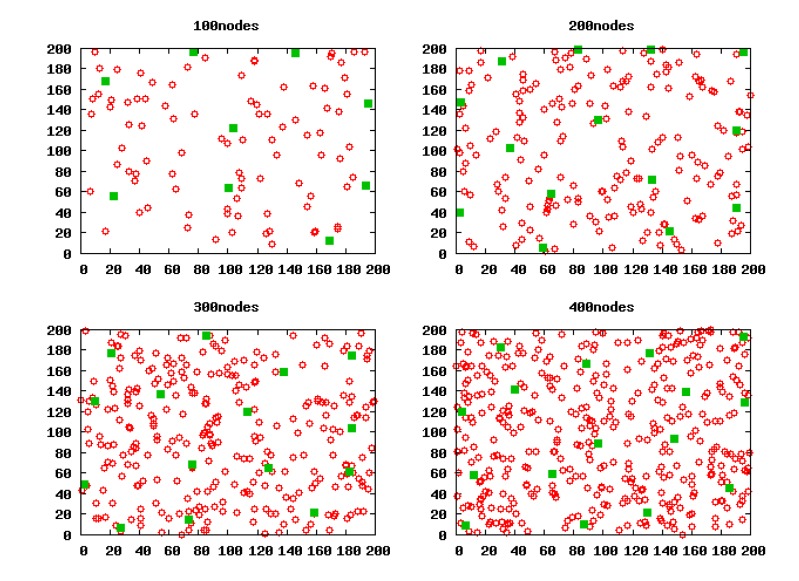
Cluster heads (CHs) generated by the local energy consumption prediction-based clustering protocol (LECP-CP).

We run LECP-CP in these scenarios, respectively. By executing the cluster head election algorithm, we can gain the selected CHs in these scenarios as shown in Figure 6. These green solid squares stand for the CHs. Obviously, in any scenario, the CHs generated by LECP-CP all distribute uniformly.

#### 7.1.1. The Number of CHs and *R_a_*

**Figure 7.**
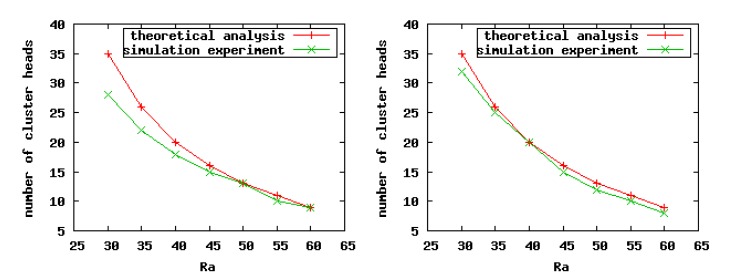
The number of CHs generated with different cluster radii *R_a_*.

As indicted in Figure 7, when we set a smaller value for the CHs' competition radius, there exists a greater difference between the theoretical analysis and simulation experiment results, especially in sparse scenarios. The reason is that the point coverage problem is converted to the area coverage problem when we carry on the theoretical analysis. Consequently, in sparse scenarios, if the competition radius of nodes is smaller, then the overlaps among clusters are less, or even none, which leads to the imprecise relationship between the number of CHs and *R_a_* in the practical experiments. For the same reason, in the dense scenarios, no matter the size of the competition radius of nodes, the theoretical analysis results are closer to the experiment results. On the whole, according to the practical simulation results, we can conclude that our theoretical analysis is correct.

#### 7.1.2. Cluster Radius *R_a_* and Network Lifetime

On network lifetime, there is no clear definition. According to the definitions given in [25], the lifetime of a WSN can be quantified using the following three kinds of metrics: (1) the time from the deployment of the network to the death of the first node (first node dies, FND); (2) the time when a certain percent of nodes are alive (percentage nodes alive, PNA); (3) the time when all of the nodes are dead in the network (last node dies, LND).

**Figure 8.**
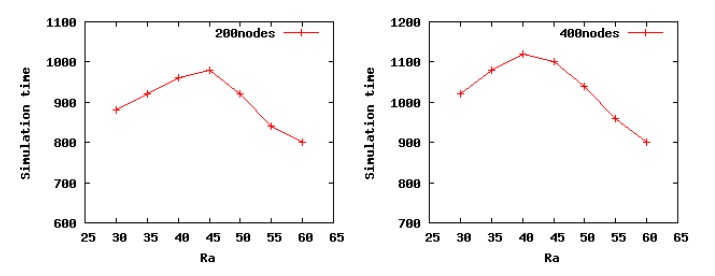
Network lifetime in Scenarios 2 and 4 when setting different values for the cluster radius *R_a_*.

Here, we define the network lifetime as the percentage nodes alive (PNA). The network lifetime is defined as the time when 90 percent of nodes are alive. To verify the conclusion obtained in Theorem 3, we choose low density Scenario 2 and high density Scenario 4 to run our algorithm. As can be seen from the Figure 8, in Scenario 2, the network lifetime increases gradually with the incrementof *R_a_* and gets its maximum value when *R_a_* is 45 m, after which, there is a slight decrease, which is consistent with our theoretical analysis results, 45.5698 m, obtained from Theorem 3. In our theoretical analysis, when the *R_a_* is set as 45.5698 m, the energy consumption of the entire network is the minimum. Furthermore, as shown in Figure 8, in Scenario 4, when *R_a_* is set as 40 m, the network lifetime achieves the maximum value, likewise, which is coincident with the optimal value 39.1604 m. On the basis of these cases, the conclusion of Theorem 3 is proven to be correct.

#### 7.1.3. CH Distribution

**Figure 9.**
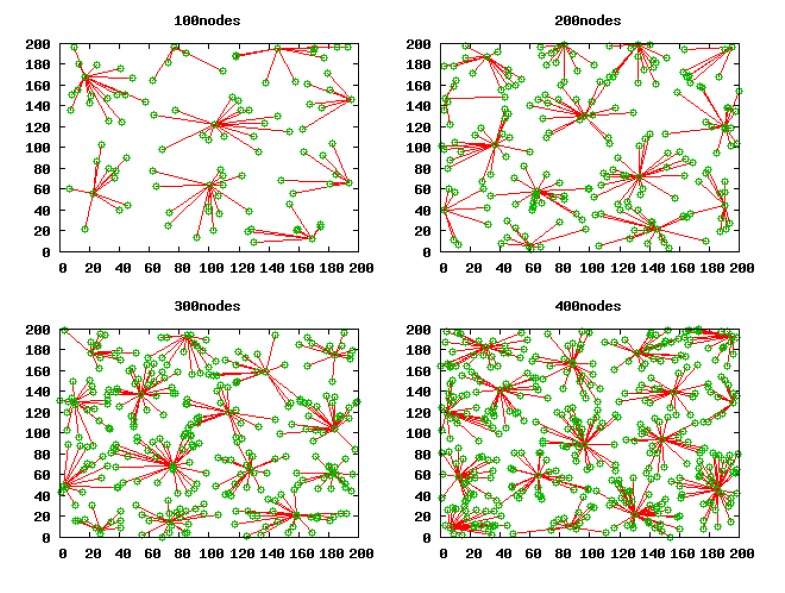
The clustering topology formed by the LECP-CP.

Based on the validity of Theorem 3, we work out the optimal cluster radius in these square scenarios with 100 nodes, 200 nodes, 300 nodes and 400 nodes, which are 55.3811 m, 46.5698 m, 42.0806 m and 39.1604 m, respectively. Then, we run LECP-CP in these scenarios in terms of these calculated cluster radii and gain the clustering topology as shown in Figure 9. Apparently, there is one and only one CH within the competition radius *R_a_* of any CH.

**Figure 10.**
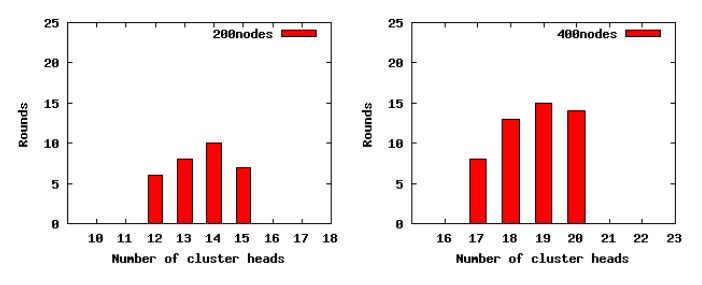
The distribution of the number of CHs.

We select 50 round results randomly to count the number of CHs generated by LECP-CP in each round. The stability analysis of the number of CHs is exhibited in Figure 10, all of which have a concentration distribution around a small interval. Thus, we can conclude that LECP-CP can achieve more stable performance on the number of CHs.

### 7.2. Network Lifetime

#### 7.2.1. Node Energy and Network Lifetime

**Figure 11.**
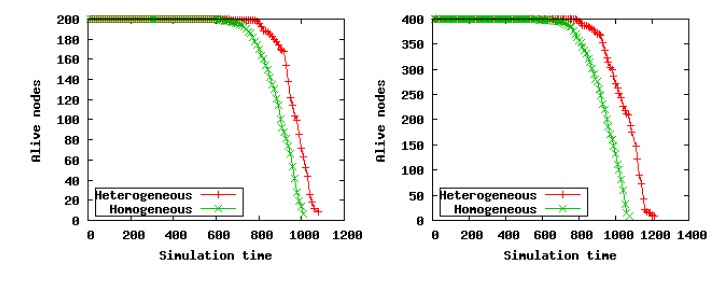
Comparison of network lifetime under heterogeneous and homogeneous scenarios.

From Figure 11, no matter if in sparse or dense scenarios, we can see that the network lifetime in heterogeneous scenarios is longer than that in homogeneous scenarios. The reason is that LECP-CP takes the local energy consumption ratio of nodes into account when selecting the CHs and routing nodes, which can take full advantage of the high-energy and low-cost nodes in heterogeneous scenarios; thus, the CHs selected are always the optimal, and thereby, the network lifetime can be extended. Thus, LECP-CP is suitable for both the heterogeneous and homogeneous scenarios.

#### 7.2.2. The Number of CHs and Network Lifetime

**Figure 12.**
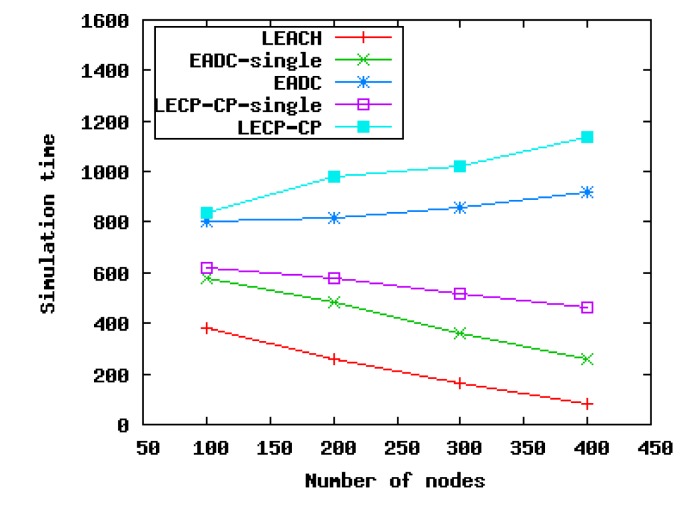
Comparison of the network lifetimes of different algorithms.

To compare with other clustering algorithms, we run LEACH, EADC and LECP-CP in different scenarios. Since CHs send the collected data to the BS directly in LEACH, we also run the cluster head election algorithms of EADC and LECP-CP, taking no account of inter-cluster communication routing, named EADC-single and LECP-CP-single, respectively. As shown in Figure 12, the network lifetimes of LEACH, EADC-single and LECP-CP-single all decrease with the increase of the node number in these networks. There are two reasons for this. On the one hand, the number of CHs generated by these algorithms is in proportion to the number of nodes in the networks; with the increase of the node number, the number of CHs, which communicate with the BS directly, also increases. On the other hand, since the control message complexity of these algorithms is *O*(*N*), where *N* is the number of nodes in these networks, thus combining these two facts together, the network lifetimes of these algorithms are definitely reduced. However, compared with LEACH and EADC-single, the network lifetime of LECP-CP-single has a less rapid decline. The reason is that LECP-CP-single can select CHs with higher residual energy and lower energy consumption; thus, CHs can save more energy for transmitting data to the BS.

**Figure 13.**
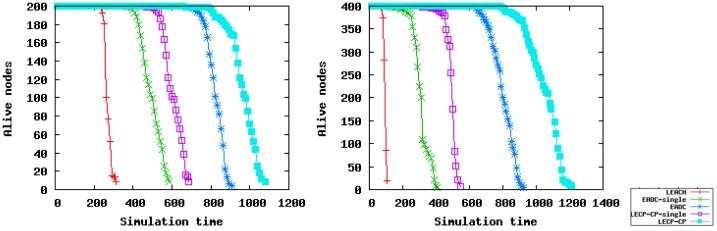
Concrete comparison of the network lifetime of different algorithms.

Taking inter-cluster communication routing into consideration is quite helpful for increasing the network lifetime, just as EADC and LECP-CP show in Figure 12. Furthermore, due to the introduction of the local energy consumption ratio, the performance of LECP-CP in the cluster setup phase and data transmission phase outperforms EADC, since the CHs generated by LECP-CP are more reasonable and the energy consumption ratio in the network is lower. Another reason is that we set the inter-cluster transmission range *R_inter–CH_* and the intra-cluster transmission range *R_intra–CH_* as *R_inter–CH_* ≥ 3*R_intra–CH_*; thus, even if the number of dead nodes becomes more and more, since *R_inter–CH_* is large enough, each connected subgraph, *i.e.*, each cluster, can still maintain a connection. CHs farther away from BS still can send their data to the BS by other forwarding CHs, instead of sending the data to the BS directly due to the lost connectivity among CHs. From this, we can conclude that LECP-CP has good scalability, and it is suitable for varying network sizes. Figure 13 shows a concrete comparison of LEACH, EADC-single, EADC, LECP-CP-single and LECP-CP in terms of network lifetime in Scenario 2 and 4, respectively. LECP-CP and EADC perform far better than LEACH, EADC-single and LECP-CP-single in prolonging network lifetime attributed to the better cluster head election algorithm and the design of the inter-cluster communication routing tree.

## 8. Conclusions

In this paper, aiming at energy heterogeneous WSNs where nodes are deployed uniformly, we propose a novel clustering protocol, LECP-CP, in which a new cluster head election algorithm is designed, which uses the predicted local energy consumption ratio of nodes as the parameter to compete for the role of the CH. Thus, the global energy consumption can be optimized by the optimization of the local energy consumption. To further reduce the energy consumption of CHs, we also propose a new inter-cluster communication routing tree construction algorithm, based on the local energy consumption ratio of nodes, as well. In addition, we provide explicit numerical calculations for the optimal cluster radius to minimize the energy consumption of the entire network, which is proven to be more accurate and realistic by theoretical analysis and simulation experiments.

## Figures and Tables

**Table 1.** Description of node state messages.

**State**	**Description**
*Candidate*	Candidate node
*Head*	Cluster head node
*Plain*	Plain node

**Table 2.** Description of control messages.

**Message**	**Description**
*Node_Msg*	Tuple(selfid, selfenergy)
*Head_Msg*	Tuple(selfid)
*Join_Msg*	Tuple(selfid, headid)
*Schedule_Msg*	Tuple(schedule, order)
*Route_Msg*	Tuple(selfid, selfratio)

**Table 3.** Parameters of the simulation.

**Parameter**	**Value**
sensor field	200 × 200
BS location	(250,100)
Number of nodes	100, 200, 300, 400
Initial energy of nodes	1–3 *J*
Data packet size	50 *nJ/bit*
*E_elec_*	50 *nJ/bit*
*∊_fs_*	10 *pJ*/(*bit*.*m*^2^)
*E_com_*	5 *nJ*/(*bit*.*signal*)
*E_sen_*	0.5 *nJ*/(*bit*.*signal*)
*DIST*	80 *m*
